# A Mixed-Methods Panel on the Uptake of New Medical Technologies for Benign Prostatic Hyperplasia in the United Arab Emirates

**DOI:** 10.7759/cureus.112558

**Published:** 2026-07-13

**Authors:** Kamran Ahmed, Ahmad Abdul-Rahman, Yassir J Mohammed, Hosam Al-Qudah, Fariborz Bagheri, Muthana Alrawi, Salam Alhasani, Thamir Alkasab, Antonia Bosworth Smith, Kim Seemann, Rhodri Saunders, Emily Woodward, Yasser Farahat

**Affiliations:** 1 Urology, Sheikh Khalifa Medical City, Abu Dhabi, ARE; 2 Urology, NMC Royal Hospital, Dubai, ARE; 3 Urology, Canadian Specialist Hospital, Dubai, ARE; 4 Urology, Al Zahra Hospital Dubai, Dubai, ARE; 5 Urology, Dubai Hospital, Dubai, ARE; 6 Urology, NMC Specialty Hospital, Dubai, ARE; 7 Urology, Mediclinic Welcare Hospital, Dubai, ARE; 8 Health Economics and Outcomes Research, Coreva Scientific GmbH and Co. KG, Königswinter, DEU; 9 Health Economics, Coreva Scientific GmbH and Co. KG, Königswinter, DEU; 10 Health Economics and Market Access, Boston Scientific Middle East FZ LLC, Dubai, ARE; 11 Urology, Sheikh Khalifa General Hospital, Umm Al Quwain, ARE

**Keywords:** benign prostatic hyperplasia, delphi panel, emerging market, lower urinary tract symptoms, water vapor thermal therapy

## Abstract

Background

Advancements in medical technologies can result in new treatment options. To better understand the complexities that clinicians and manufacturers navigate when new medical technologies are introduced in the United Arab Emirates (UAE), water vapor thermal therapy for benign prostatic hyperplasia (BPH) was used as a case study.

Methods

This mixed-methods Delphi study included a panel of nine expert urologists practicing in the UAE. Two anonymous online surveys were completed, followed by an in-person meeting. Consensus statements were developed and graded using a nine-point scale, where 1.0 indicated complete agreement, and 9.0 indicated complete disagreement.

Results

Thirteen consensus statements were developed. After two rounds of voting, a strong consensus (range: 1.1-1.8) was reached for all statements. It emerged that no single technology was the best for all patients with BPH (score: 1.2) and that the generation of local data (score: 1.2) and Gulf region-specific adaptation of international guidelines (score: 1.6) would facilitate the integration of new technologies in the UAE. The panel stressed the need for manufacturers and suppliers to work closely with local authorities to support clinician and patient education (score: 1.2). Statements related to reimbursement strategies were also discussed.

Conclusions

The study highlights the importance of regional adaptation of international guidelines, physician and patient education, and collaboration in emerging markets. The panel emphasized patient-specific decision-making, access to treatment, and the role of data-driven reimbursement pathways. Future studies should address regional data gaps to refine strategies for adopting new medical technologies in the UAE and other emerging markets.

## Introduction

Benign prostatic hyperplasia (BPH) is an enlargement of the prostate (>30 mL) caused by a nonmalignant overgrowth of prostatic tissue around the urethra [1]. This enlargement may lead to lower urinary tract symptoms (LUTS) and even bladder outlet obstruction [1]. Although BPH is not life-threatening, it may cause symptoms that affect quality of life, including urinary urgency, nocturia, frequent urinary tract infections, and sexual dysfunction [2,3]. These symptoms can substantially affect a patient’s physical and mental health and significantly reduce quality of life [3,4]. A 2022 study reported a 19.7% prevalence of BPH among men >50 years of age in the United Arab Emirates (UAE) [3]. Meanwhile, the prevalence of BPH-associated LUTS in this age group (approximately 11.9-31.7%) has been reported only collectively for the Gulf cluster countries [3,5].

In the UAE, there is an increased demand for advanced health care services and cutting-edge medical devices [6]. The health care system is evolving across all sectors. These aspects of the health care infrastructure, coupled with economic constraints, different incentives for payers and providers, and the specific needs of the local population, create a shifting landscape that can increase the complexity of introducing medical technologies [6]. Water vapor thermal therapy (WVTT) has emerged as a minimally invasive surgical procedure for treating LUTS due to BPH because it offers advantages such as preservation of erectile and ejaculatory function [7]. An initial study conducted at a hospital in Dubai found WVTT to be safe and effective for the treatment of moderate and severe LUTS [8].

There is a need to identify the factors that clinicians and manufacturers should consider when a new minimally invasive surgical technology is introduced into emerging markets such as the UAE [9,10]. The aim of this Delphi study was to better understand these factors, using WVTT for the treatment of LUTS due to BPH as a case study, with the key outcome being the generation of consensus.

## Materials and methods

Study type

This was an observational mixed-methods study. The Delphi technique was selected for its ability to effectively address questions that benefit from a structured synthesis of expert judgment [11]. The Delphi panel included nine experienced urologists currently practicing in the UAE. This panel size conformed to the typical Delphi panel size requirements (8-23 experts) for the health care sector [11]. As this study did not determine statistical significance between outcomes, no power or sample size calculation was performed. The moderator (RS) was experienced in this methodology.

Setting and Delphi panel selection

In line with the good practice framework [12], the panelists were selected based on predefined criteria, including professional qualifications, clinical experience, and expertise in BPH management. The inclusion criteria for the panelists were >5 years of BPH experience, use of BPH treatment technology, treatment of >10 patients with BPH per month, membership in the UAE Urology Association, and a scientific publication record in the field. Candidates with low awareness of WVTT were excluded. The panelists were aware that this study required continued commitment throughout all steps of the Delphi process [13].

Study process

**Figure 1:**
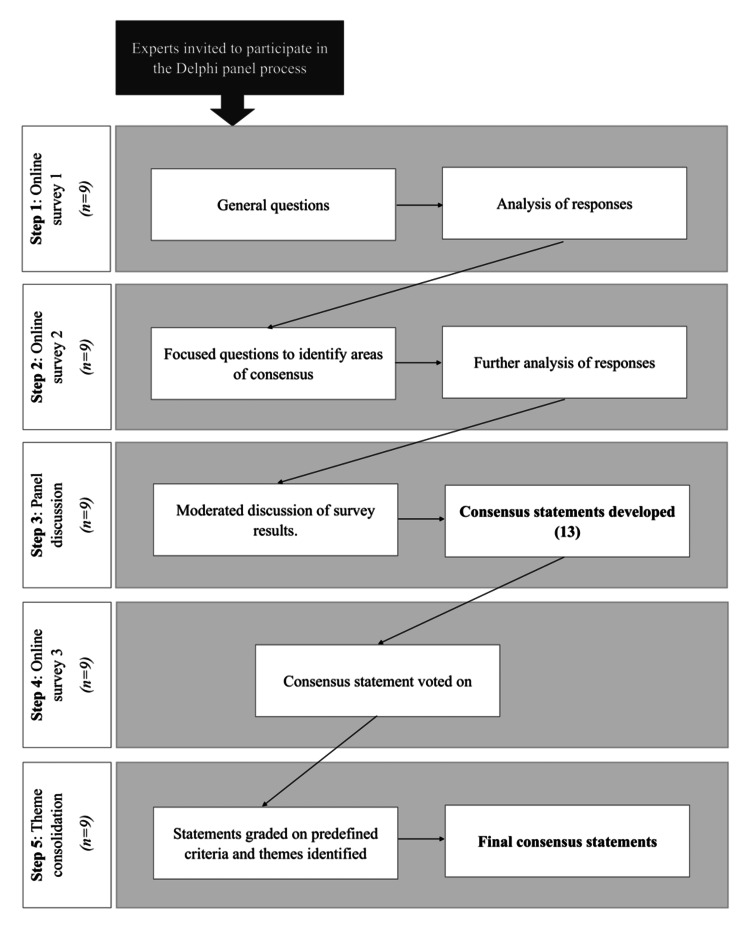
Five-step Delphi process This figure was created using Microsoft PowerPoint (Microsoft Corporation, Redmond, WA, USA).

**Figure 2:**
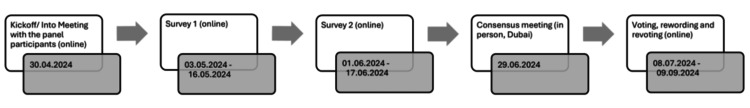
Detailed timeline of the Delphi process This figure was created using Microsoft PowerPoint (Microsoft Corporation, Redmond, WA, USA).

**Figure 3:**
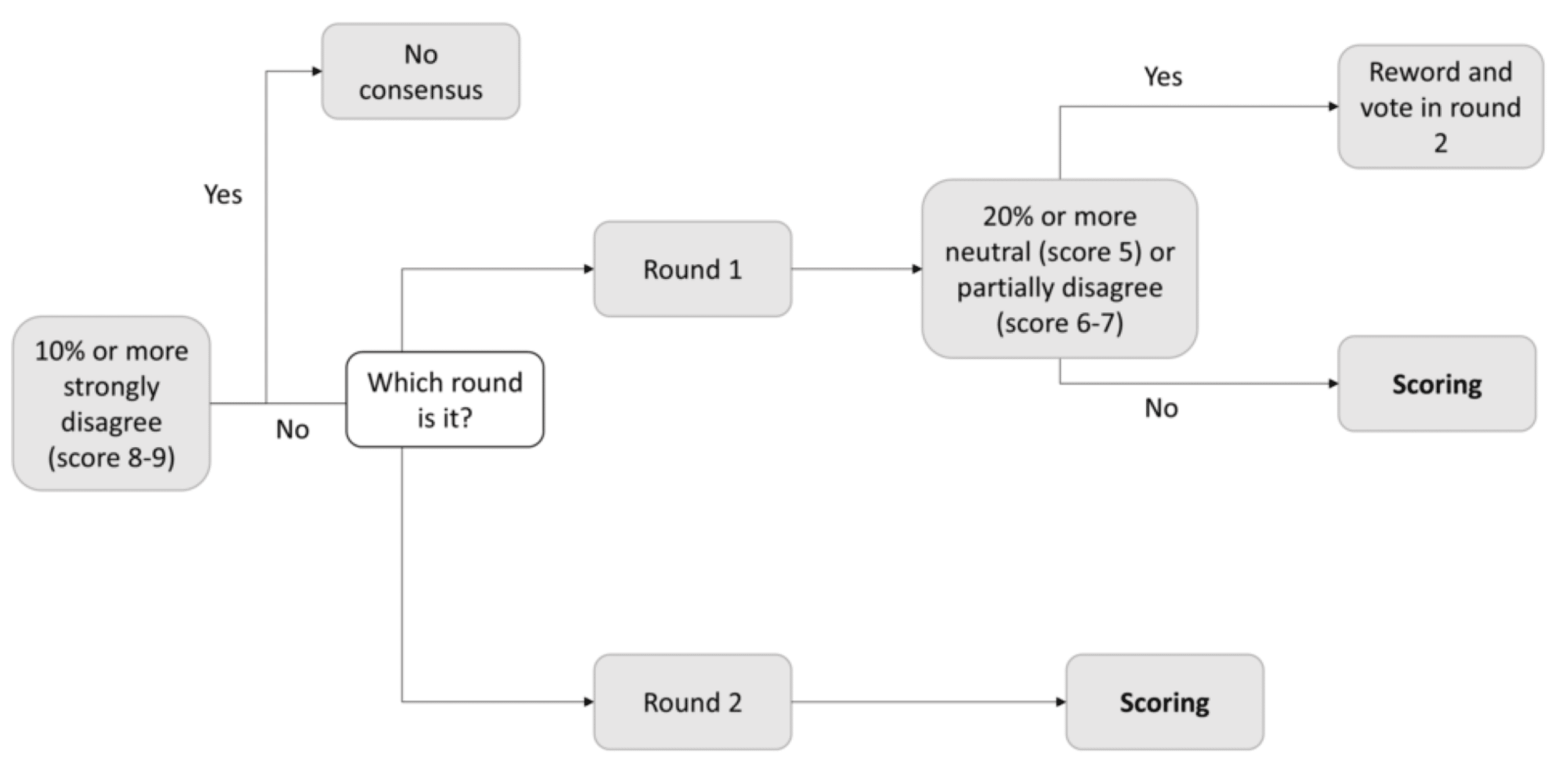
Decision tree representation of the consensus development and selection processes The scale was predefined by the team conducting the panel (KS, RS, and ABS) before the start of the Delphi process. The scoring ranged from 1 (strongly agree) to 9 (strongly disagree). This figure was created using Microsoft PowerPoint (Microsoft Corporation, Redmond, WA, USA).

A five-step Delphi process was conceptualized and executed by the research team (Figure 1). The process began in March 2024 and continued until September 2024 (Figure 2). The panel completed two anonymous online surveys (steps 1 and 2) and attended one in-person meeting (step 3). During the five-hour in-person meeting held in Dubai in June 2024, the panel discussed the results of the two surveys. Consensus statements were then drafted. After the meeting, the panelists voted anonymously online on the final consensus statements (step 4). Voting was based on a predefined nine-point scale ranging from “strongly agree” (scores 1-2) and “partially agree” (scores 3-4) to “neutral” (score 5), “partially disagree” (scores 6-7), and “strongly disagree” (scores 8-9). For all statements with a ≥20% rate of “neutral” or “partially disagree” responses, rewording was offered, followed by a round of revoting. If ≥10% of the panelists strongly disagreed with a statement, the statement was considered not to have reached consensus (Figure 3). Once voting was completed, the statements were graded based on predefined criteria during the theme consolidation round (step 5) to determine the level of consensus, ranging from 1.0 (“strong consensus”) to 9.0 (“no consensus”).

Surveys

Survey 1 (19 questions) covered the following topics: panelists’ backgrounds, experience treating BPH, treatments offered, practices used in the panelists’ hospitals, general access to BPH treatment in the UAE, and patient profiles for WVTT.

Survey 2 (15 questions) used Likert scales and ranking questions to categorize the responses gathered from Survey 1 and identify potential topics for consensus statement development. The survey covered the following topics: clinical factors affecting decision-making, patient preferences, BPH treatment options offered in the panelists’ hospitals, costs and reimbursement procedures, patient selection for WVTT, and the guidelines used for BPH treatment and patient selection. Both surveys were administered via the LimeSurvey platform (LimeSurvey GmbH, Hamburg, Germany). The surveys are provided in Appendix A and Appendix B. Statistical summaries of the survey results were generated by KS and ABS using Microsoft Excel (Microsoft Corporation, Redmond, WA, USA).

Ethics and compliance

Survey data were anonymized, minutes were recorded during the focus group meeting, and all data were stored in a GDPR-compliant manner. The study sponsor is a manufacturer of WVTT technology. The sponsor was involved in the study conceptualization, logistics for the in-person consensus meeting, and manuscript review. They were not involved in the development of the consensus statements, nor did they participate in the surveys or the consensus meeting. The study protocol and associated contracts were reviewed by the Health Media Lab Institutional Review Board and granted an exemption (approval 2506).

## Results

Panel composition

Nine urology experts practicing in the UAE participated in the study (Figure 1). The panelists had between 11 and 30 years of clinical experience (weighted mean: 21 years) and represented a mix of university (2), government (2), and private hospitals (7) (some panelists were affiliated with more than one type of hospital). Most participants reported high volumes of BPH cases at their institutions, with 55% treating >40 BPH cases per month, and had experience with numerous LUTS treatment modalities. The most frequently used technologies were WVTT, bipolar transurethral resection of the prostate, prostatic urethral lift, photoselective vaporization of the prostate, bipolar enucleation of the prostate, and transurethral incision of the prostate.

Consensus generation

After completing the online surveys, the experts attended an in-person meeting, which led to the development of 13 consensus statements (Table 1). All statements reached a strong consensus after the final round of voting. The voting scale ranged from 1 (strongly agree) to 9 (strongly disagree). The statements were subsequently grouped into the following major themes: (1) Guidelines and local data; (2) Optimizing personalized treatment; (3) Physician education and collaboration; (4) Reimbursement strategy for new devices; and (5) Experience with WVTT. The voting distribution for each statement is included in Appendix C.

**Table 1:** Consensus statements relating to the introduction of WVTT for the treatment of BPH-associated LUTS grouped into five themes BPH, benign prostatic hyperplasia; LUTS, lower urinary tract symptoms; WVTT, water vapor thermal therapy

Statement		Score	Strength
Guidelines and local data			
1	It would be beneficial to develop an adaptation of international guidelines as the standard for the Gulf region and surrounding areas to better serve the local population.	1.6	Strong
2	To inform best-practice decisions, more long-term data on interventions for LUTS due to BPH are needed. Preferably, these would include local data on: (a) outcomes and adverse events; (b) reintervention rates; and (c) the type and effectiveness of any reintervention. We advocate stratifying these data by prostate size.	1.2	Strong
Optimizing personalized treatment			
3	There is no single optimal intervention for every case, and new interventions do not necessarily replace existing interventions but instead provide additional options that can optimize patient care.	1.2	Strong
4	Based on clinical factors alone, there are often multiple suitable interventions for patients with LUTS due to BPH. The initial responsibility of clinicians is to determine all suitable interventions that would benefit the patient.	1.2	Strong
5	We recommend counseling patients on the pros and cons of each suitable intervention for LUTS due to BPH, allowing patient preference to be incorporated into the decision-making process.	1.2	Strong
6	To improve shared decision-making, we encourage the development of accessible patient education materials through trusted local authorities, associations, and societies.	1.3	Strong
Physician education and collaboration			
7	Once an intervention has been agreed upon by the patient and physician, it should be performed by a qualified and experienced surgeon, even if that means referral to another provider.	1.1	Strong
8	We encourage manufacturers and suppliers to collaborate with local authorities, associations, and societies to support physician education and training, thereby ensuring an understanding of new medical devices on the market.	1.2	Strong
Reimbursement strategy for new devices			
9	To increase access to new medical devices, a procedure-specific reimbursement code approved by the local authorities would be beneficial. Discussions between the hospital and insurer are needed to determine appropriate reimbursement.	1.4	Strong
10	We encourage manufacturers and suppliers to work with local authorities, hospitals, and clinicians to provide evidence on the cost-benefit of new medical devices.	1.3	Strong
11	When considering the cost-benefit of a new medical device, hospitals should evaluate the balance between capital costs and the cost per case. The cost per case includes running, consumable, and indirect costs (e.g., operating room time, training, and length of stay).	1.3	Strong
Experience with WVTT			
12	From our clinical experience, when the following patient characteristics are present, we encourage physicians to also explore alternatives to WVTT: (a) chronic prostatitis; (b) narrow/high bladder neck; (c) urinary retention/high postvoid residual; and (d) dual bladder outlet obstruction (stricture).	1.8	Strong
13	WVTT is an appropriate option for patients who are being managed with medication and who would like to discontinue medication, are noncompliant, or are nonresponders. These patients have mild to moderate symptoms.	1.3	Strong

Guidelines and local data

The panelists reported using international guidelines, including those published by the European Association of Urology [14,15], the American Urological Association [16], and the National Institute for Health and Care Excellence for the management of BPH-associated LUTS [17]. However, the panelists emphasized the need for local data that better represent the UAE population, given differences in the prevalence and characteristics of BPH between the UAE and Western countries. Furthermore, tailoring guidelines to reflect the unique health care environment and population characteristics of the UAE was highlighted as a priority.

Optimizing personalized treatment

All panelists agreed that no single treatment was best for all patients; instead, treatment should be tailored to each patient’s characteristics and clinical needs. Based on these factors, more than one treatment option may be suitable. The panel emphasized that new technologies should complement rather than replace existing treatments; as such, they should provide additional options or address gaps in care to further optimize patient outcomes. In addition to clinical considerations, the experts acknowledged that external factors considerably influence treatment choices. Patients often arrive at consultations with predetermined treatment preferences. The panelists agreed that it is the physician’s responsibility to present all suitable treatment options to patients, even if their own facility does not offer them. Furthermore, long-term data on the treatment of LUTS due to BPH are needed to demonstrate sustained symptom improvement over extended periods and to guide appropriate treatment selection.

Physician education and collaboration

The panelists recommended that manufacturers collaborate with local authorities, associations, or societies to ensure that physicians are fully informed about newly available technologies and treatments. The information provided should cover the indications and contraindications for a specific treatment, the correct use of the technology, and any potential side effects or adverse events associated with its use. Given that new technologies are associated with a learning curve [18], planning physician training before technology adoption is paramount.

The panelists stressed the value of increasing patient awareness of new and existing treatment options. The dissemination of reliable and accessible information, together with efforts to dispel misinformation, will support the shared decision-making process.

Reimbursement strategy for new devices

The panelists agreed that, during the introduction of a new medical device, it is crucial to ensure that patients have access to treatment. One substantial barrier to widespread adoption by health care providers is cost. The panelists agreed that obtaining a specific reimbursement code for a new procedure, such as WVTT, would help ensure that hospitals are fairly reimbursed for the treatment provided. Moreover, the panelists emphasized that hospitals must carefully consider the balance between the capital cost of a device (i.e., purchase price, installation cost, infrastructure adjustments, and initial training) and its cost per case (i.e., the cost associated with using the device for each procedure). Some technologies may have a higher capital cost but reduce the cost per case over time by, for instance, decreasing operating room time, staffing costs, and patient length of hospital stay. By taking a comprehensive approach to cost evaluation, hospitals can better justify the acquisition of new technology.

Experience with WVTT

The panelists identified specific patient groups for whom WVTT was especially appropriate. These included patients with mild to moderate symptoms who were dissatisfied with their existing medication because of nonresponse, nonadherence, or the daily burden and cost of taking medication. WVTT was also noted as a favorable option for younger patients or those wishing to start a family because this technique offers better preservation of erectile and ejaculatory function than other treatment alternatives [7]. When considering the balance between immediate symptom relief and sustained long-term outcomes, WVTT was considered a compelling option for patients who preferred a minimally invasive procedure and were willing to wait longer for symptom resolution. In addition to identifying patient groups well suited for WVTT, the panelists outlined patient characteristics that may warrant caution when recommending WVTT. These include chronic prostatitis, a narrow or high bladder neck, urinary retention, high postvoid residual urine, and dual bladder outlet obstruction.

## Discussion

This Delphi study explored the key issues surrounding the introduction of new medical technologies into the UAE market, using the uptake of WVTT for the treatment of BPH-associated LUTS as a case study. To the best of our knowledge, this is the first Delphi study to investigate this topic. The panel of experts reached strong consensus on all 13 statements developed (Table 1). They concluded that the successful introduction of new technologies into the UAE requires: (1) the generation of new clinical guidelines based on local data; (2) the optimization of patient selection and treatment selection; (3) increased physician and patient education and collaboration; (4) a unified reimbursement strategy for new devices; and (5) the simultaneous adoption of new technology by multiple institutions.

International guidelines are still widely used in the UAE and other countries of the Gulf Cooperation Council because no uniform guidelines exist for LUTS treatment in this region. This lack of local guidelines was illustrated by the panelists’ use of international guidelines. The panel emphasized the need for more local data to inform the adaptation of international guidelines, noting that differences in disease prevalence and health care infrastructure necessitate a region-specific approach. Gulf countries have experienced a substantially greater increase in BPH cases than Western countries in recent years, with a 110-500% versus a 38% increase in case volume reported for the 2000-2019 period [19]. These findings reflect differences in population growth, age structure, and health care access that are not accounted for in international guidelines.

The recommended regional adaptation of international urology guidelines is expected to take some time, as sufficient data are needed to accurately capture and account for demographic and clinical differences between the UAE population and those of the countries from which existing guidelines were derived. Encouragingly, however, UAE-specific data on new BPH treatment technologies are beginning to emerge [8,20]. For instance, two retrospective observational studies performed at Thumbay University Hospital (Ajman, UAE) [20] and Mediclinic City Hospital (Dubai, UAE) [8] reported that WVTT was effective for treating LUTS due to BPH. Further local research will play a crucial role in shaping clinical guidelines that address the unique needs of the UAE population. Although this adaptation process requires time and resources, it represents an important step toward optimizing patient care and ensuring that guidelines are relevant and effective.

The panelists agreed that patient preference should be considered during treatment selection; however, they also emphasized the importance of working with the patient to select the most appropriate treatment option. Situations in which suboptimal treatments are selected based on patient preference alone should be avoided. The nine panelists strongly agreed that, although local patient selection guidelines are important, treatment should be optimized according to the characteristics of each patient. Thus, although clinical guidelines are undeniably valuable, the panel agreed that they should remain adaptable rather than overly rigid. A recent case study of a 56-year-old patient in the UAE illustrates how patient preference can influence treatment decisions, stressing the importance of collaborative decision-making between physicians and patients [21]. Furthermore, a narrative review of WVTT concluded that treatment decisions are best made through shared decision-making, with consideration of the needs and characteristics of each individual [9].

As the primary users of new medical technology, health care professionals must develop a thorough understanding of each device’s intended use and functionality. Learning curves are a well-documented factor in the successful adoption of new medical technologies [18], including WVTT [9]. The panel identified the importance of physician and patient education for the successful adoption of new technologies. Implementing comprehensive initial training sessions and ongoing educational initiatives to keep physicians up to date with best practices can be vital to the uptake of new technology.

LUTS can have a profound effect on patients’ mental health [4,22]. Thus, in addition to addressing the physical aspects of LUTS, clinicians must be aware of the psychological burden of this condition and its role in appropriate treatment selection [23]. Comprehensive patient education on how LUTS can affect mental health and quality of life can also help patients better understand their condition. In addition to developing appropriate educational materials for patients, efforts should be made to dispel misinformation related to a given treatment. Collaboration among health care institutions, local authorities, and insurance providers could facilitate the development of these educational materials.

Patient access to innovative treatments such as WVTT depends heavily on economic and regulatory factors. For instance, it is important that hospitals are fairly compensated by payers for adopting new medical technologies, as this can considerably affect access. The UAE, like many countries, has general reimbursement codes for medical procedures and devices; however, new and emerging treatments such as WVTT often require the development of specific codes, which can take time. The UAE is in the process of implementing Current Procedural Terminology (CPT) codes (as used in the United States) and, to our knowledge, is not diverging from this established system at present. However, as regional practices evolve, it may become necessary to develop local coding and reimbursement processes to ensure that regulations remain agile and applicable to local circumstances. The panelists agreed that establishing specific reimbursement codes could be beneficial; however, this represents only one aspect of a broader strategy.

When entering an emerging market, manufacturers need to be aware that perceptions of costs, as well as the terminology used, may differ among stakeholders. A clear dialogue should be established with all stakeholders before market entry to ensure that appropriate incentives are in place and that the technology is implemented in a way that delivers clear value across the health care system. Differences in insurance systems among the emirates and a diverse mix of health care providers can complicate the integration and standardization of new technologies. Despite these challenges, the UAE is making a concerted effort to unify licensing, digitize health records, and harmonize strategies to streamline the adoption of innovative technology across the country. For instance, the recent introduction of “Riayati,” a National Unified Medical Record (NUMR) system, and its integration with the “Malaffi” (Abu Dhabi Health Data Services) and “Nabidh” (Dubai Health Information Exchange) platforms promise to streamline patient referral processes and unify the health care sector [24].

Examining the introduction of new medical technologies into emerging markets offers valuable insights. The present case study revealed that the initial launch of WVTT in the UAE was successful, with the device appealing to both patients and physicians. However, to maximize its potential, interventions should be applied selectively and appropriately. The panel also agreed that, although WVTT offered clear benefits, especially for patients with milder forms of disease who may not require more invasive treatment, long-term studies are needed to validate its efficacy and safety in the diverse patient population of the UAE. This view has also been expressed by experts from the wider Middle East region [25]. Such longitudinal research would also help refine patient selection criteria and optimize treatment pathways, enabling the effective dissemination of this technology across the UAE. For now, clinicians in the UAE must largely rely on findings from long-term follow-up studies conducted outside the country [26]. However, once the technology has been in use for some time, usage guidelines can be refined and adjusted based on local experience.

Strength and limitations

The strength of the present study lies in its integration of qualitative and quantitative methods to explore the complex issues that arise during the market entry of new technologies. This flexible approach enabled the inclusion of insights from physicians actively using the technology in clinical practice.

We acknowledge that this study also had several limitations. Although the size of the expert panel aligned with recommendations for Delphi studies in the health sciences and reflected the size of the UAE population, it was relatively small; therefore, the generalizability of the findings cannot be guaranteed. Despite our efforts to include panelists from diverse clinical settings, most practiced in private hospitals, which may limit the applicability of the findings regarding reimbursement, access, and technology adoption across the broader UAE health care system. Thus, the transferability of the findings across institutions and health care settings should be considered at the reader’s discretion. As this is a single-technology case study in urology, factors influencing the introduction of new technologies may differ in other specialties. A further limitation is that the study focused exclusively on physician perspectives. Incorporating the views of additional stakeholders (e.g., payers, hospital administrators, and regulatory bodies) could have yielded valuable insights, particularly regarding reimbursement and access. Similarly, including patient representatives would have better reflected patient needs, preferences, and values. These gaps present opportunities for future research. Finally, as an observational study, this work may be subject to selection bias. The findings should therefore be interpreted in light of these limitations and the study methods, particularly those described in the Ethics and compliance section.

Future research

Future studies could investigate the impact of physician perspectives on policy and reimbursement decisions in the UAE. It would also be informative to explore the views of other relevant stakeholders.

## Conclusions

The Delphi panel reached consensus on 13 statements relating to clinical, educational, financial, and patient-centered factors that may be relevant to the implementation of WVTT for LUTS due to BPH in the UAE. Our findings suggest that access to emerging therapies in the UAE could potentially be improved by integrating local data into international guidelines, optimizing personalized treatment, enhancing physician and patient education, and addressing reimbursement barriers. However, these findings should be interpreted in the context of the study limitations. Future research incorporating a broader range of stakeholders, including payers, hospital administrators, regulatory bodies, and patients, would help validate these findings.

## Appendices

Appendix A: Survey 1

Background

To get started, we will be asking you about your general workplace setup, clinical specialty, and clinical experience.

1. Which of these best describes your current workplace(s)? (multiple selection)

University hospital

Public/government hospital

Private (all insured patients)

Private (only higher-tier insured patients)

Other

2. Which of these best describes your work experience/previous workplace(s)? (multiple selection)

University hospital

Public/government hospital

Private (all insured patients)

Private (only higher-tier insured patients)

Other

3. How would you describe your clinical speciality? (open)

4. How many years of experience in your clinical speciality do you have? (single answer question)

1-5 years

6-10 years

11-20 years

21-30 years

31-40 years

Experience with benign prostate hyperplasia (BPH)

In this section, we will be asking you about your experience with treating BPH and the considerations for treatment selection.

5. How many BPH cases do you treat in a month? (single answer question)

Less than 5

5-10

11-20

21-30

31-40

More than 40

6. Which guidelines do you refer to when considering management for BPH? (single answer question)

Local hospital guidelines

American Urological Association (AUA)

European Association of Urology (EAU)

Department of Health Abu Dhabi (DoH)

Dubai Health Authority (DHA)

National Institute for Health and Care Excellence (NICE)

I do not follow a specific guideline

Other (please specify)

7. How important are these clinical factors when selecting a BPH treatment plan? (Please rate all options) (Please rate all options on a scale of 1 being very important to 7 being not important at all)

i. Age

ii. Comorbidities (e.g., Charlson Comorbidity Index)

iii. History of bothersome prostate symptoms

iv. International Prostate Symptom Score (IPSS)

v. Prostate volume

vi. Noninvasive urodynamics (urine flow cytometry)

vii. Previous treatments (surgical or pharmacological)

viii. Preservation of sexual function (IIEF)

ix. Preservation of fertility

8. Are there any other clinical factors that you consider when selecting a BPH treatment plan?

Yes

No

9. If yes, could you please elaborate on the other clinical factors you would consider?

10. In addition to guidelines and clinical factors, which of these other factors do you consider when selecting a BPH treatment plan? (multiple answers possible, select all that apply)

i. Patient co-pay

ii. Resource use

iii. Scheduling and availability

iv. Time to treatment

v. Cost

vi. Reimbursement

vii. Previous experience with certain treatment options

viii. Patient preference

ix. Environmental impact

x. Patient insurance

xi. Insurance approval

xii. Financial constraints

xiii. Other (specify)

Treatment options in your hospital

This section explores what BPH treatments are used in your hospital.

11. What surgical treatment interventions for BPH are on offer in your hospital? (multiple answers possible, select all that apply)

Monopolar transurethral resection of the prostate (M-TURP)

Bipolar transurethral resection of the prostate (B-TURP)

Bipolar enucleation of the prostate

Transurethral incision of the prostate (TUIP)

Photoselective vaporization of the prostate (PVP)

Vapo-enucleation

Green laser enucleation

Water vapor thermal therapy (WVTT)

Temporary implanted prostatic device (TIPD)

Prostatic stent

Prostatic urethral lift (PUL)

Holmium laser enucleation (HoLEP)

Thulium laser enucleation of the prostate (ThuLEP) or thulium fiber laser enucleation of prostate (ThufLEP) or thulium fibre enucleation (ThufibreLEP)

Other

12. Ranking question based on previous answers: Please rank these from most frequently used to least frequently used in your hospital. (ranking all selected answers from Q11)

13. In your opinion, why is (position 1 from Q12) the most frequently performed treatment for BPH in your hospital? (open - 100 words)

14. In your opinion, why is (last position from Q12) not as frequently performed as a treatment for BPH in your hospital? (open)

If section, depending on answers Q11 and Q12.

15. In your opinion, why is water vapor treatment less frequently used than (position 1 from Q12) for BPH? (open), If Q11, water vapor is selected, and Q12 water vapor is neither top nor last.

16. In your opinion, why is water vapor treatment not routinely offered in your hospital as a treatment option for BPH? (open). If Q11 does not, select water vapor.

Access to BPH treatment in the UAE

This section explores access and differences in hospital settings in the UAE

17. Do you feel that the treatment plan that you have described previously would also apply to other hospital types in the UAE? (Yes/No/I don’t know)

18. If not, please describe some of the key differences that you anticipate. (open)

Patient profiles

19. Please describe a patient to whom you would prescribe WVTT. (open)

Appendix B: Survey 2

Scientific team

Clinical factors in decision-making

In Survey 1, we asked you and your peers about the following clinical factors:

Age

Comorbidities

History of bothersome prostate symptoms

International Prostate Symptom Score (IPSS)

Prostate volume 

Non-invasive urodynamics (urine flow cytometry)

Previous treatments (surgical or pharmacological)

Preservation of sexual function (IIEF)

Preservation of fertility

All these factors were considered important in forming your treatment decision. All respondents, however, mentioned that there were additional factors that needed to be considered. The following question explores these additional factors mentioned by you and your peers in Survey 1:

1. How important are these additional clinical factors when selecting a BPH treatment plan? Please rank up to 5 of the most important factors in your decision-making.

a. Bladder diverticulum

b. Bladder stones

c. Coagulopathy and blood thinner use

d. Dual bladder outlet obstruction (stricture, detrusor dysfunction)

e. Hematuria

f. Hydronephrosis

g. Narrow bladder neck

h. Patient expectations/patient preference

i. Presence of a prostatic median lobe

j. Prostate-specific antigen (PSA)

k. Recurrent UTI

l. Renal impairment

m. Special occupation

n. Urinary retention/high postvoid residual

o. Uroflowmetry

Patient preference

Patient preferences and expectations were frequently reported as being important in decision-making.

2. How important to your patients, on a scale of 1 being very important to 7 being not important at all, are the following factors?

Same question for different age groups (< 40 years, 40-60 years, and >60 years)

a. Reduction in postoperative pain

b. Shorter hospital stay

c. Level of co-pay

d. Preservation of ejaculation or reduced ejaculatory dysfunction

e. Preservation of erectile function

f. Time to resolution of symptoms

g. Need for a catheter

Treatment options in your hospital

From Survey 1, we know that the following four treatment options for BPH are the most commonly used by you and your peers.

Bipolar transurethral resection of the prostate

Prostatic urethral lift

Transurethral incision of the prostate

WVTT

3. Where are these procedures most frequently performed in your hospital? (single answer)

a. Inpatient procedure not performed in the operating room

b. Inpatient procedure performed in the operating room

c. Outpatient procedure/day case

d. Not performed in my hospital

4. Please rank the treatment options based on the following criteria:

a. Highest cost to the hospital

b. Highest preservation of ejaculation function

c. Highest preservation of erectile function

d. Longest length of hospital stay

e. Longest time to alleviation of symptoms

f. Best long-term outcomes

5. Would you like to increase or decrease the use of certain treatment options, but are restricted by other factors apart from clinical factors and patient preference? (Yes/No)

6. If yes, please briefly describe factors restricting your choice. (open answer)

Costs and reimbursement

In Survey 1, the majority selected costs as an important factor in selecting a BPH treatment. However, there was agreement that reimbursement does not play a role in decision-making.

7. Please briefly explain how costs impact you in your decision-making. (open answer)

8. Are there any incentives, institutional policies, or other system dynamics that restrict or promote the utilization of specific treatment options? (Yes/No)

9. If yes, please give a short description. (open answer)

10. In a hypothetical situation, where there are two possible BPH treatments: Tx-A and Tx-B that are similar in safety and efficacy, but with different operating room utilization. If the hospital wanted to encourage use of Tx-B, which of the following would be the most appropriate strategy? (single answer)

a. Working with the authorities to obtain a dedicated reimbursement code covering the cost of the Tx-B device and the procedure

b. Demonstrating to the insurance company the cost and patient benefits of using Tx-B over Tx-A

c. Any other strategies

11. Please explain why. (open answer)

Patient selection for WVTT

12. The following patient characteristics were identified as favorable for selecting WVTT as a treatment for BPH. To what extent do you agree that each factor is favorable for selecting WVTT as the treatment option? (scale: 1 = strongly agree to 7 = strongly disagree. The order of the response options is randomized for each participant.)

Acceptance of post-procedure side effects

Age

LUTS/IPSS severity

Prostate volume

Wants to preserve sexual activity

Wants to preserve ejaculation function

Post-void residual <200ml

Normal prostate-specific antigen levels

Absence of urinary tract infection

No median lobe

Guidelines

In Survey 1, we asked about the guidelines that your hospital uses. All respondents reported using international guidelines such as NICE, AUA, and EUA.

13. Should the local/ regional guidelines be updated? (Yes/No)

14. If yes, please briefly suggest key areas of the local/regional guidelines that should be updated.

General

15. If you feel that it is relevant, please provide us with details of any important papers or evidence that everyone needs to be aware of and that could be discussed during the in-person meeting. (open, not mandatory)

Appendix C: Consensus scoring distribution

**Table 2:** Distribution of scoring per statement

Score	1	2	5
Statement 1	7	1	1
Statement 2	7	2	0
Statement 3	7	2	0
Statement 4	7	2	0
Statement 5	7	2	0
Statement 6	6	3	0
Statement 7	8	1	0
Statement 8	7	2	0
Statement 9	5	4	0
Statement 10	6	3	0
Statement 11	6	3	0
Statement 12	6	3	0
Statement 13	5	3	1

## References

[1] Roehrborn CG (2005). Benign prostatic hyperplasia: an overview. Rev Urol.

[2] Al Shareef Z, Al-Shahrabi R, Saheb Sharif-Askari F (2023). Risks of prostate cancer and mortality in the city of Sharjah, United Arab Emirates. Front Oncol.

[3] Noweir A, Abusamra A, Al Zarooni A (2022). Prevalence of benign prostatic hyperplasia among the adult general population of five Middle Eastern Countries: Results of the SNAPSHOT programme. Arab J Urol.

[4] Pinto JD, He HG, Chan SW, Toh PC, Esuvaranathan K, Wang W (2015). Health-related quality of life and psychological well-being in patients with benign prostatic hyperplasia. J Clin Nurs.

[5] Arafa MA, Farhat K, Aqdas S, Al-Atawi M, Rabah DM (2015). Assessment of lower urinary tract symptoms in Saudi men using the International Prostate Symptoms Score. Urol Ann.

[6] Howard JJ (2014). Medical devices and the Middle East: market, regulation, and reimbursement in Gulf Cooperation Council states. Med Devices (Auckl).

[7] Arezki A, Sadri I, Couture F (2021). Reasons to go for Rezūm steam therapy: an effective and durable outpatient minimally invasive procedure. World J Urol.

[8] Rowaiee R, Akhras A, Lakshmanan J, Sikafi Z, Janahi F (2021). Rezum therapy for benign prostatic hyperplasia: Dubai's initial experience. Cureus.

[9] Kaltsas A, Giannakodimos I, Symeonidis EN (2025). To Rezūm or not to Rezūm: a narrative review of water vapor thermal therapy for benign prostatic hyperplasia. J Clin Med.

[10] Yang J, Wu W, Amier Y (2023). Efficacy and safety of water vapor thermal therapy in the treatment of benign prostate hyperplasia: a systematic review and single-arm meta-analysis. BMC Urol.

[11] Shang Z (2023). Use of Delphi in health sciences research: a narrative review. Medicine (Baltimore).

[12] Jorm A (2025). Steps in carrying out a Delphi study. Using the Delphi Method to Establish Expert Consensus. Advancing Methods for Interdisciplinarity in the Social Sciences.

[13] Hasson F, Keeney S, McKenna H (2000). Research guidelines for the Delphi survey technique. J Adv Nurs.

[14] Gravas S, Gacci M, Gratzke C (2023). Summary paper on the 2023 European Association of Urology Guidelines on the management of non-neurogenic male lower urinary tract symptoms. Eur Urol.

[15] (2024). Management of non-neurogenic male LUTS. https://uroweb.org/guidelines/management-of-non-neurogenic-male-luts.

[16] Sandhu JS, Bixler BR, Dahm P, Goueli R, Kirkby E, Stoffel JT, Wilt TJ (2024). Management of lower urinary tract symptoms attributed to benign prostatic hyperplasia (BPH): AUA guideline amendment 2023. J Urol.

[17] (2010). Lower urinary tract symptoms in men: management. https://www.nice.org.uk/guidance/cg97.

[18] Govindarajulu US, Stillo M, Goldfarb D, Matheny ME, Resnic FS (2017). Learning curve estimation in medical devices and procedures: hierarchical modeling. Stat Med.

[19] (2022). The global, regional, and national burden of benign prostatic hyperplasia in 204 countries and territories from 2000 to 2019: a systematic analysis for the Global Burden of Disease Study 2019. Lancet Healthy Longev.

[20] Abouelgreed TA, Koritenah AK, Badran Y (2023). Evaluation of Rezum therapy as a minimally invasive modality for management of benign prostatic hyperplasia: a prospective observational study. Arch Ital Urol Androl.

[21] Rowaiee R, Akhras A, Janahi FK (2021). Rezum therapy, a feasible and safe treatment for the larger prostate. Int J Surg Case Rep.

[22] Zhang X, Ma L, Li J, Zhang W, Xie Y, Wang Y (2024). Mental health and lower urinary tract symptoms: results from the NHANES and Mendelian randomization study. J Psychosom Res.

[23] Chan AS, Chan SW, Estivalet AG (2023). Mitigating lower urinary tract symptoms secondary to benign prostatic hyperplasia: ameliorating sexual function and psychological well-being in older men. Am J Mens Health.

[24] UAE Health Authorities announce successful integration between “Riayati”. https://mohap.gov.ae/en/media-center/news/31/1/2023/uae-health-authorities-announce-successful-integration-between-riayati-malaffi-and-nabidh.

[25] Azhar RA, Elkoushy M, Subahi M (2024). Awareness and compliance of urologists in the Middle East with minimally invasive surgical devices for the management of benign prostate hyperplasia. Urol Ann.

[26] McVary KT, El-Arabi A, Roehrborn C (2021). Preservation of sexual function 5 years after water vapor thermal therapy for benign prostatic hyperplasia. Sex Med.

